# Expression patterns and prognostic relevance of subtype‐specific transcription factors in surgically resected small‐cell lung cancer: an international multicenter study

**DOI:** 10.1002/path.5922

**Published:** 2022-05-25

**Authors:** Zsolt Megyesfalvi, Nandor Barany, Andras Lantos, Zsuzsanna Valko, Orsolya Pipek, Christian Lang, Anna Schwendenwein, Felicitas Oberndorfer, Sandor Paku, Bence Ferencz, Katalin Dezso, Janos Fillinger, Zoltan Lohinai, Judit Moldvay, Gabriella Galffy, Beata Szeitz, Melinda Rezeli, Christopher Rivard, Fred R Hirsch, Luka Brcic, Helmut Popper, Izidor Kern, Mile Kovacevic, Jozef Skarda, Marcel Mittak, Gyorgy Marko‐Varga, Krisztina Bogos, Ferenc Renyi‐Vamos, Mir Alireza Hoda, Thomas Klikovits, Konrad Hoetzenecker, Karin Schelch, Viktoria Laszlo, Balazs Dome

**Affiliations:** ^1^ Department of Thoracic Surgery Semmelweis University and National Institute of Oncology Budapest Hungary; ^2^ National Koranyi Institute of Pulmonology Budapest Hungary; ^3^ Department of Thoracic Surgery, Comprehensive Cancer Center Medical University of Vienna Vienna Austria; ^4^ 1st Department of Pathology and Experimental Cancer Research Semmelweis University Budapest Hungary; ^5^ Department of Physics of Complex Systems Eotvos Lorand University Budapest Hungary; ^6^ Department of Pathology Medical University of Vienna Vienna Austria; ^7^ MTA‐SE NAP, Brain Metastasis Research Group Hungarian Academy of Sciences Budapest Hungary; ^8^ Torokbalint County Institute of Pulmonology Torokbalint Hungary; ^9^ Division of Oncology, Department of Internal Medicine and Oncology Semmelweis University Budapest Hungary; ^10^ Department of Biomedical Engineering Lund University Lund Sweden; ^11^ Division of Medical Oncology University of Colorado Anschutz Medical Campus Aurora CO USA; ^12^ Tisch Cancer Institute, Center for Thoracic Oncology Mount Sinai Health System New York NY USA; ^13^ Diagnostic and Research Institute of Pathology Medical University of Graz Graz Austria; ^14^ University Clinic for Respiratory and Allergic Diseases Golnik Golnik Slovenia; ^15^ Institute of Clinical and Molecular Pathology, Medical Faculty Palacky University Olomouc Olomouc Czech Republic; ^16^ Department of Pathology University Hospital Ostrava and Faculty of Medicine University of Ostrava Ostrava Czech Republic; ^17^ Department of Surgery University Hospital Ostrava and Faculty of Medicine University of Ostrava Ostrava Czech Republic; ^18^ Department of Thoracic Surgery Klinik Floridsdorf Vienna Austria

**Keywords:** small cell lung cancer, molecular subtypes, prognostic relevance, expression pattern, immunohistochemistry, ASCL1, NEUROD1, POU2F3, YAP1, neuroendocrine subtypes

## Abstract

The tissue distribution and prognostic relevance of subtype‐specific proteins (ASCL1, NEUROD1, POU2F3, YAP1) present an evolving area of research in small‐cell lung cancer (SCLC). The expression of subtype‐specific transcription factors and P53 and RB1 proteins were measured by immunohistochemistry (IHC) in 386 surgically resected SCLC samples. Correlations between subtype‐specific proteins and *in vitro* efficacy of various therapeutic agents were investigated by proteomics and cell viability assays in 26 human SCLC cell lines. Besides SCLC‐A (ASCL1‐dominant), SCLC‐AN (combined ASCL1/NEUROD1), SCLC‐N (NEUROD1‐dominant), and SCLC‐P (POU2F3‐dominant), IHC and cluster analyses identified a quadruple‐negative SCLC subtype (SCLC‐QN). No unique YAP1‐subtype was found. The highest overall survival rates were associated with non‐neuroendocrine subtypes (SCLC‐P and SCLC‐QN) and the lowest with neuroendocrine subtypes (SCLC‐A, SCLC‐N, SCLC‐AN). In univariate analyses, high ASCL1 expression was associated with poor prognosis and high POU2F3 expression with good prognosis. Notably, high ASCL1 expression influenced survival outcomes independently of other variables in a multivariate model. High POU2F3 and YAP1 protein abundances correlated with sensitivity and resistance to standard‐of‐care chemotherapeutics, respectively. Specific correlation patterns were also found between the efficacy of targeted agents and subtype‐specific protein abundances. In conclusion, we investigated the clinicopathological relevance of SCLC molecular subtypes in a large cohort of surgically resected specimens. Differential IHC expression of ASCL1, NEUROD1, and POU2F3 defines SCLC subtypes. No YAP1‐subtype can be distinguished by IHC. High POU2F3 expression is associated with improved survival in a univariate analysis, whereas elevated ASCL1 expression is an independent negative prognosticator. Proteomic and cell viability assays of human SCLC cell lines revealed distinct vulnerability profiles defined by transcription regulators. © 2022 The Authors. *The Journal of Pathology* published by John Wiley & Sons Ltd on behalf of The Pathological Society of Great Britain and Ireland.

## Introduction

Small‐cell lung cancer (SCLC) remains one of the most lethal forms of malignant diseases [1, 2, 3]. Unlike the increasingly personalized approach in non‐SCLC (NSCLC) treatment, SCLC is still regarded clinically as a molecularly homogeneous disease with a single histological type [3, 4]. Although RB1 and P53 protein expression might have clinical importance in surgically treated NSCLC patients [5], the biallelic losses of *RB1* and *TP53* (the genomic hallmark of SCLC) are so prevalent in SCLC that they cannot define subclasses [1, 6, 7]. However, recent SCLC profiling studies of both human tumors and preclinical models (such as SCLC cell lines, genetically engineered mouse models, and patient‐derived xenografts) suggest distinct SCLC subtypes defined by the relative expression of four key transcriptional regulators: ASCL1 (SCLC‐A), NEUROD1 (SCLC‐N), POU2F3 (SCLC‐P), and YAP1 (SCLC‐Y) [4].

ASCL1‐high tumors have been reported to be associated with elevated expression of neuroendocrine (NE) markers, whereas NEUROD1‐high tumors with lower overall NE marker expression and, therefore, with a less NE phenotype [4, 8, 9, 10, 11, 12]. With regard to non‐NE subtypes, the divergent expression profile and transcription factor dependency of POU2F3‐high tumors suggest that the SCLC‐P subtype may arise from a distinct cell of origin and might represent a specific tuft‐cell variant of SCLC [4, 13]. YAP1, a transcriptional regulator in the Hippo signaling pathway, is suspected to be preferentially expressed in a subset of non‐NE SCLC as well [4, 14], although subsequent immunohistochemical (IHC) analyses failed to confirm a unique YAP1‐driven subtype in human tissue samples [12]. Importantly, *in silico* and preclinical analyses have also questioned the existence of a distinct YAP1 subtype and proposed a unique triple‐negative subtype accompanied by an inflamed gene signature (SCLC‐I) [15]. Of note, the link between SCLC molecular subtypes and P53/RB1 protein expression is still largely unexplored.

Analyses of both human SCLC tumors and murine SCLC models revealed that most tumors harbor substantial intratumoral heterogeneity with regard to the expression pattern of subtype‐specific transcription regulators [6, 15, 16, 17]. Aspects of this heterogeneity might be implicated in tumor behavior and therapeutic resistance, and might be of diagnostic importance when classifying the patients according to the dominant molecular subtype of their tumor [18]. Molecular expression patterns may be thus more evident in surgical specimens than in small SCLC biopsy samples, and the dominant subtype can be more accurately recognized in surgical samples [18, 19]. SCLC is very rarely resected surgically [20], and thus whole SCLC tissue blocks are generally not available [6]. Accordingly, only a few studies have been conducted so far to investigate the tissue expression of subtype‐specific transcription factors by IHC in surgically resected SCLC tissue specimens [12, 21, 22]. Sato *et al* [21] reported the presence of four key transcriptional regulators in only 47 surgically resected SCLC samples, whereas Baine *et al* [12] assessed the expression of these markers and analyzed their associated histologic characteristics in a mixed cohort of 43 primary tumor resections, 105 biopsies, and 26 fine‐needle aspirates. Qu *et al* [22] investigated the associations between subtype‐specific proteins and NE differentiation markers by using tissue microarrays (TMAs). Importantly, due to the low number of cases with whole tissue sections of surgically resected tumors, the lack of detailed survival data and the pronounced intratumoral heterogeneity of SCLC, these prior studies warrant further validation concerning in particular the clinicopathological and prognostic relevance of subtype‐defining proteins.

In the present multicenter study, we investigated the expression pattern, clinical significance, and prognostic relevance of subtype‐specific transcription factors (ASCL1, NEUROD1, POU2F3, and YAP1), as well as P53 and RB1 proteins in a large cohort of surgically treated SCLC patients comprising 386 samples. Additionally, in order to unfold the correlation patterns between subtype‐specific proteins and *in vitro* efficacy of targeted and chemotherapeutic agents, we also performed a comprehensive mass spectrometry (MS)‐based proteomic analysis in a panel of 26 human SCLC cell lines.

## Materials and methods

### Study population and treatment

In this multicenter study, we included 386 patients with histologically confirmed SCLC who underwent surgical resection in five Central European medical centers. The study was conducted in accordance with the guidelines of the Helsinki Declaration of the World Medical Association and with the approval of the national level Ethics Committee of each participating country. Due to the retrospective nature of the study, the requirement for written informed consent was waived. After clinical information was collected, patient identifiers were removed, and, subsequently, patients could not be identified either directly or indirectly. Further details of the study population and treatments are provided in Supplementary materials and methods.

### Patient samples and immunohistochemistry

We grouped the patients either into a *whole tissue section (WTS)* cohort where complete surgical formalin‐fixed paraffin‐embedded (FFPE) blocks were available (*n* = 141) or a *TMA* cohort (tissue microarray cohort, *n* = 247) and analyzed these two cohorts by IHC separately. TMA and IHC protocols are described in detail in Supplementary materials and methods.

### Proteomic analyses and *in vitro* cell viability assays

In total, 26 commercially available human SCLC cell lines were subjected to in‐depth proteomic analyses. The *in vitro* efficacy of therapeutic agents was assessed by determining their corresponding IC_50_ values in each cell line. Details concerning MS‐based proteomic analyses and cell viability assays are described in Supplementary materials and methods.

### Statistical analyses

All statistical analyses were performed using R version 3.6.3 (R Foundation for Statistical Computing, Vienna, Austria). See Supplementary materials and methods for details.

## Results

### Patient and sample characteristics

A total of 141 surgically resected SCLC patients were included in the *WTS* cohort, whose clinicopathological characteristics are summarized in supplementary material, Table S2. The median age of included patients was 63.9 years (range: 41–83). All individuals had a Caucasian background and 85 of them were male (60.7%). With regard to the expression pattern of subtype‐specific proteins, we found that patients with high ASCL1‐ and NEUROD1‐expressing tumors tended to have late‐stage disease at diagnosis, whereas POU2F3 expression was nonsignificantly associated with early‐stage SCLC (supplementary material, Table S2). Moreover, when analyzing the *WTS* cohort, we also found that intratumoral necrosis is a feature of low NEUROD1‐expressing tumors. No statistically significant associations were found between P53 or RB expression and clinicopathological characteristics. The *TMA* cohort consisted of 245 SCLC patients. Although these patients also underwent lung resection surgery, in their case only TMA specimens were available (supplementary material, Table S3). The median age of patients in the *TMA* cohort was 57 years (range, 37–79 years) and the included patients were predominantly male (76.4%). Of note, due to the relatively long inclusion period, clinicopathological data of the *TMA* cohort was not available in some of the cases (supplementary material, Table S3). We found no statistically significant associations between the expression pattern of key transcription factors and clinicopathological characteristics in the *TMA* cohort. Nevertheless, a similar (yet statistically not significant) tendency was observed in the case of ASCL1 expression and tumor stage as in the *WTS* cohort. Accordingly, the majority of late‐stage SCLC patients had high ASCL1‐expressing tumors in the *TMA* cohort (supplementary material, Table S3). As for the antibodies used for quality check of the TMA samples, we found strong positivity with Bcl‐2 [23, 24] and INI1 [25], and moderate positivity with Ki‐67 [26, 27] and SYP [28] (supplementary material, Figure S1).

### Molecular subtypes of surgically resected SCLC tissue samples

**Figure 1:**
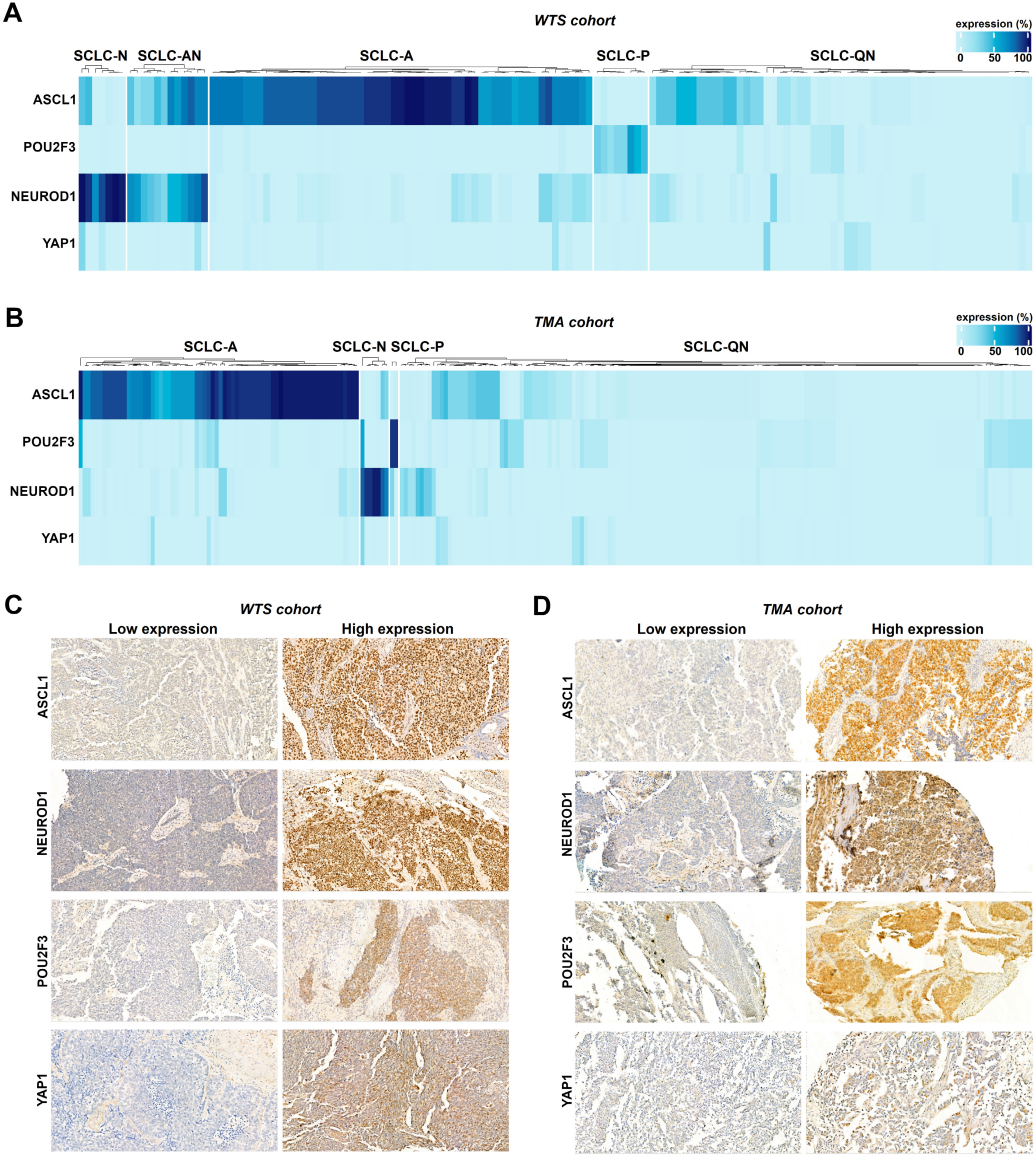
Molecular subtypes of WTS and TMA samples of surgically resected SCLCs defined by the IHC expression of ASCL1, NEUROD1, POU2F3, and YAP1. (A) Unsupervised clustering of the WTS cohort revealed five distinct SCLC subgroups. In addition to SCLC‐N (NEUROD1‐dominant), SCLC‐AN (combined ASCL1/NEUROD1), SCLC‐A (ASCL1‐dominant), and SCLC‐P (POU2F3‐dominant), we found a fifth, quadruple‐negative SCLC subtype (SCLC‐QN) with low ASCL1, NEUROD1, POU2F3, and YAP1 expressions. Clustering was performed using the R statistical computing environment, and the color bar scale represents the IHC expression level of the transcription factors as a percentage of tumor cells showing positive staining. (B) Four major clusters were identified in the TMA cohort by unsupervised hierarchical clustering defined by the expression pattern of ASCL1, NEUROD1, POU2F3, and YAP1: SCLC‐A, SCLC‐N, SCLC‐P, and SCLC‐QN. (C,D) IHC staining of representative tumors from (C) the WTS and (D) the TMA set, demonstrating the expression pattern for each transcription factor. All images were captured with a 40× objective lens.

Differential expression of the key transcription regulators clearly distinguished five major SCLC subtypes in the *WTS* cohort (Figure 1A). The expression levels for unsupervised hierarchical clustering were used as continuous variables. As shown in Figure 1A, besides SCLC‐A (ASCL1‐dominant), SCLC‐AN (combined ASCL1/NEUROD1), SCLC‐N (NEUROD1‐dominant), and SCLC‐P (POU2F3‐dominant), cluster analyses identified a fifth, quadruple‐negative SCLC subtype (SCLC‐QN) characterized by the low expression of all four investigated transcription factors. Pathologically, two manifestation forms of intratumoral heterogeneity were seen in the *WTS* cohort. In some tissue specimens, subtype‐specific marker‐expressing and nonexpressing cells appeared in a mixed form within a tumorous area, whereas in other cases clusters of these cells were found in spatially truly distinct regions.

Importantly, except for the SCLC‐AN subtype, the presence of all major subtypes distinguished in the *WTS* cohort was confirmed in the *TMA* cohort (Figure 1B). Notably, no unique YAP1 subtype was distinguished by IHC analyses in either cohort. Representative images of high versus low subtype‐specific marker expressions in WTS and TMA specimens are shown in Figure 1C,D, respectively.

### Correlation patterns of subtype‐specific transcription factor and P53 and RB1 expression in surgically resected SCLC

**Figure 2:**
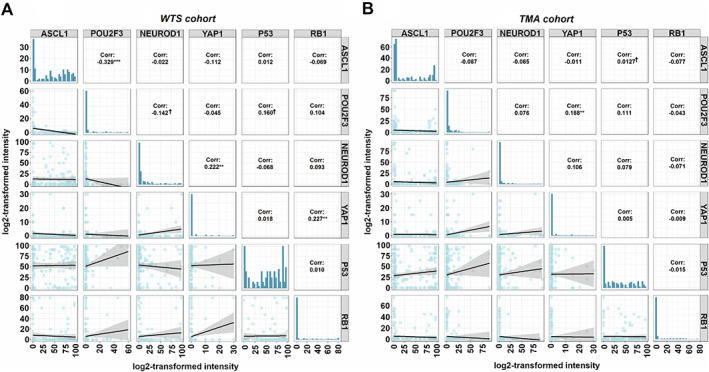
Correlation patterns of ASCL1, NEUROD1, POU2F3, YAP1, P53, and RB1 proteins in surgically resected SCLC. (A) Scatterplot showing a statistically significant positive linear correlation in the *WTS* cohort between YAP1 and NEUROD1 (*R* = 0.222) and between YAP1 and RB1 (*R* = 0.227). ASLC1 and POU2F3 expressions are significantly negatively correlated (*R* = −0.329). (B) A statistically significant positive linear correlation between YAP1 and POU2F3 expression (*R* = 0.188) in the *TMA* cohort. Correlation coefficients indicate the Pearson *r*‐values, whereas the characters following these values indicate the level of significance (***p < 0.001; **p < 0.01; *p < 0.05; †*p* < 0.10).

By analyzing the *WTS* cohort, a statistically significant weakly positive linear correlation was found between YAP1 and NEUROD1 (*r* = 0.222), and moreover between expression of YAP1 and RB1 (*r* = 0.227) (Figure 2A). Of note, however, YAP1 expression was rarely seen either in the *WTS* or in the *TMA* cohort (supplementary material, Figure S2). Therefore, all results concerning YAP1 expression should be interpreted with caution. Additionally, we also observed a moderate negative linear correlation between expression of ASCL1 and POU2F3 (*r* = −0.329; Figure 2A). Notably, we found no significant correlation between P53 and subtype‐specific protein expression in the *WTS* cohort. In the *TMA* cohort, no statistically significant results were found except for a weak positive correlation between YAP1 and POU2F3 (*r* = 0.188; Figure 2B).

### Subtype‐specific proteins serve as prognostic markers in surgically resected SCLC

**Figure 3:**
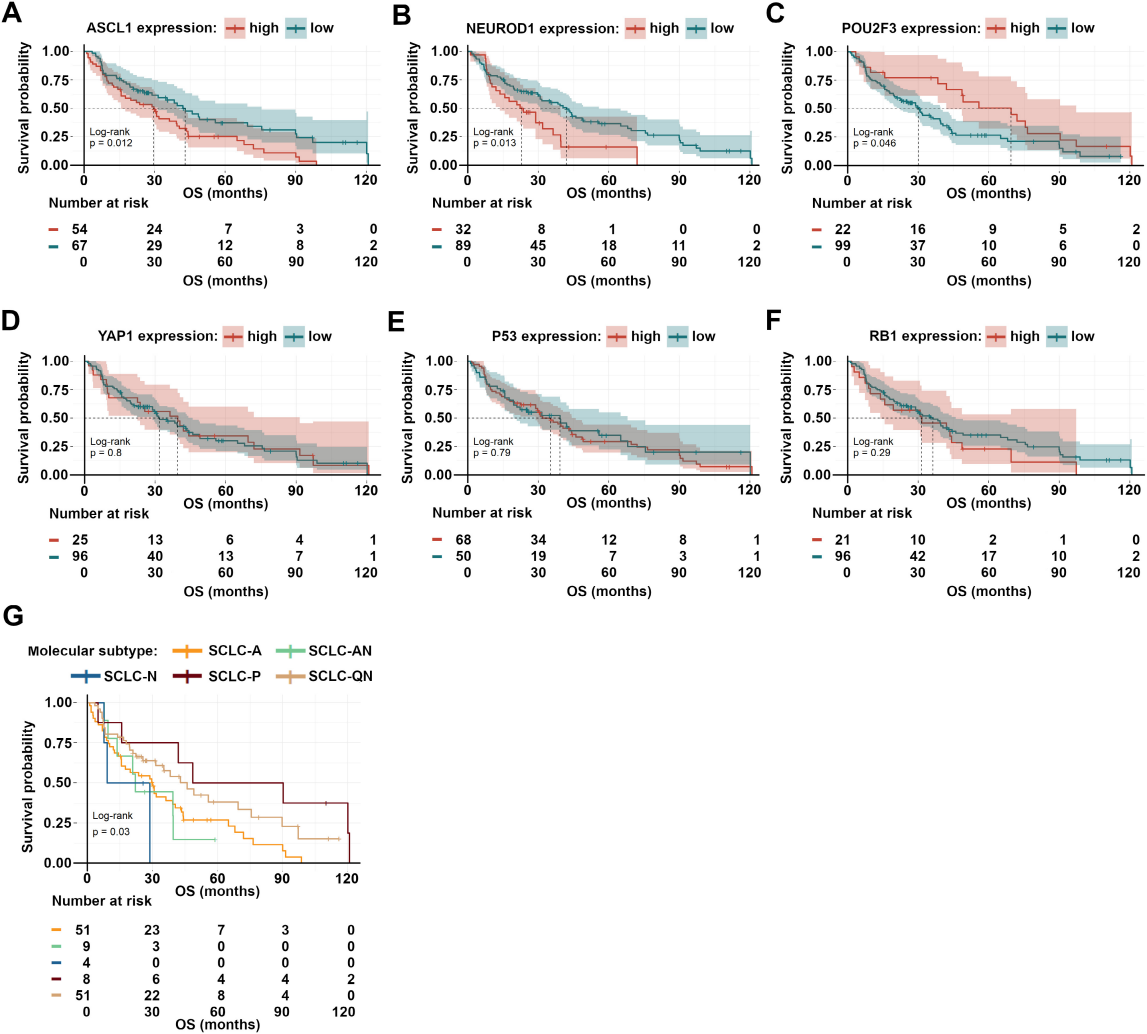
Kaplan–Meier estimates for OS in surgically treated SCLC patients according to the expression of subtype‐specific transcription factors and P53 and RB1 in the *WTS* cohort. (A) Patients with high ASCL1‐expressing tumors exhibited significantly worse median OS than those with low ASCL1‐expressing SCLCs (*p* = 0.012). (B) High NEUROD1 expression conferred significantly shorter OS (versus low NEUROD1 expression; *p* = 0.013). (C) SCLC patients with high POU2F3‐expressing tumors had significantly improved OS (versus those with low POU2F3 expression; *p* = 0.046). (D–F) YAP1, P53, and RB1 expressions did not have any impact on OS. (G) According to the dominant molecular subtypes, SCLC‐P and SCLC‐QN were associated with improved survival, whereas SCLC‐A, SCLC‐N, and SCLC‐AN with impaired survival (*p* = 0.031). Differences between different groups were compared using the log‐rank test. The cutoff values used to dichotomize patients into low and high subgroups were 50% for ASCL1, 5% for NEUROD1, 1% for POU2F3, positivity (>0%) for YAP1, 50% for P53, and positivity (>0%) for RB1.

The median follow‐up time for patients in the *WTS* cohort was 58.9 months, whereas the median overall survival (OS) was 35.3 months. First, we performed a univariate survival analysis in order to identify the clinical prognostic factors for OS (supplementary material, Figure S3). As expected, we found that patients who received adjuvant chemotherapy (CHT) after surgery exhibited significantly improved OS compared to CHT‐naïve patients (*p* = 0.00027, supplementary material, Figure S3J). Anatomic resection as a surgical approach also conferred significantly longer OS (versus wedge resection surgery; *p* = 0.056, supplementary material, Figure S3G). There were no significant associations between OS and gender (supplementary material, Figure S3A) or histological features (such as intratumoral necrosis and vascular invasion, supplementary material, Figure S3H,I). Notably, we found that high ASCL1 expression was associated with impaired survival outcomes in surgically resected patients (versus low ASCL1 expression; median OSs were 29.63 versus 42.93 months, respectively; *p* = 0.012; Figure 3A and supplementary material, Table S4). Patients with high NEUROD1‐expressing tumors also had significantly shorter OS (versus those with low NEUROD1 expression; median OSs were 22.88 versus 41.93 months, respectively; *p* = 0.013, Figure 3B and supplementary material, Table S4). In contrast, in our univariate model, high POU2F3 expression was significantly associated with improved OS (versus low POU2F3 expression, median OSs were 69.47 versus 30.07 months, respectively; *p* = 0.046, Figure 3D and supplementary material, Table S4). Next, we grouped the patients according to their tumors' dominant molecular subtype (Figure 1A). As expected, the highest survival rates were found in SCLC‐P and SCLC‐QN, and the lowest in SCLC‐A, SCLC‐N, and SCLC‐AN subtypes (*p* = 0.03; Figure 3G and supplementary material, Table S4). Accordingly, the NE phenotype proved to be a sign of poor prognosis in surgically resected SCLC (*p* = 0.003; supplementary material, Figure S4).

**Figure 4:**
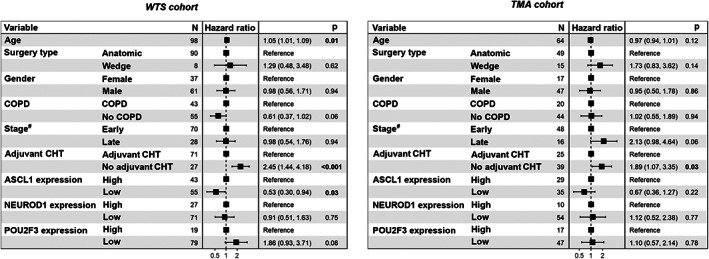
Multivariate Cox regression model for clinicopathological variables influencing the OS in the *WTS* and *TMA* cohort*s* of surgically resected SCLCs. In the *WTS* cohort, older age and high ASCL1 expression were statistically significant negative prognostic factors for OS, whereas adjuvant CHT was associated with improved survival outcomes. Cox regression analysis also revealed that patients in the *WTS* cohort with high POU2F3‐expressing tumors have a clinically relevant tendency for better survival (*p* = 0.08). Concordance of the multivariate model = 67%. In the *TMA* cohort, low ASCL1 expression and high POU2F3 expression tended to associate with better survival. Concordance of the multivariate model = 69%. OS, overall survival; CHT, chemotherapy; COPD, chronic obstructive pulmonary disease. ^#^Early‐stage refers to stage I and II, whereas late‐stage to stage III and IV SCLC.

In order to assess if the prognostic value of ASCL1, NEUROD1, and POU2F3 expression was independent of other variables (such as disease stage or therapeutic approaches) in the *WTS* cohort, we performed a multivariate Cox regression analysis (Figure 4). The model was adjusted for clinical factors such as age, gender, chronic obstructive pulmonary disease (COPD), tumor stage at diagnosis, and treatment. We found that high ASCL1 expression remained a significant negative prognosticator for OS (*p* = 0.03; Figure 4). Nevertheless, despite the elevated hazard ratios with borderline significance (*p* = 0.08) detected in patients with high POU2F3‐expressing tumors, POU2F3 expression did not influence the survival outcomes independently of other clinicopathological variables (Figure 4). As expected, age (*p* = 0.01) and adjuvant CHT (*p* < 0.001) independently influenced the OS. NEUROD1 expression had no significant impact on survival in our multivariate model.

**Figure 5:**
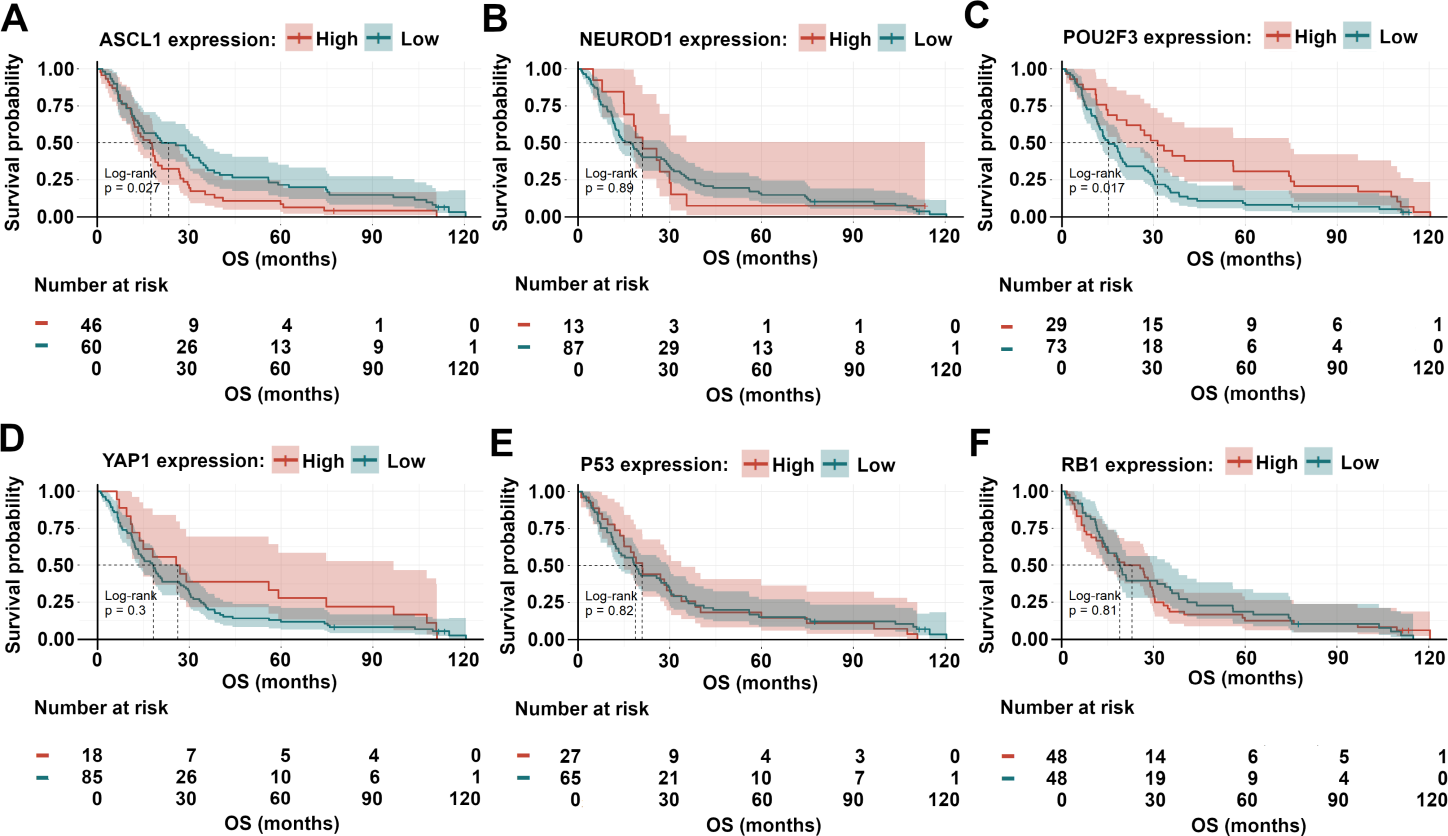
Kaplan–Meier curves for OS in surgically treated SCLC patients according to the expression of subtype‐specific proteins and P53 and RB1 in the *TMA* cohort. (A) High ASCL1 expression was associated with significantly shorter median OS (versus low ASCL1 expression; *p* = 0.027). (B) The OS did not differ significantly between patients with low versus high NEUROD1 tumor expression (*p* = 0.89). (C) Patients with high POU2F3‐expressing tumors had significantly better OS than those with low POU2F3‐expressing SCLCs (*p* = 0.017). (D–F) YAP1, P53 and RB1 expression did not have any impact on OS. Differences between different groups were compared using the log‐rank test. The cutoff values used to dichotomize patients into low and high subgroups were 5% for ASCL1, 5% for NEUROD1, 1% for POU2F3, positivity (>0%) for YAP1, 50% for P53, and positivity (>0%) for RB1.

In the *TMA* cohort, the median follow‐up time was 113.3 months, while the median OS was 18.8 months. By using univariate survival analysis (supplementary material, Figure S5), we identified significantly longer OS in patients with early‐stage disease (versus late‐stage SCLC; *p* < 0.0001), adjuvant CHT (versus adjuvant CHT‐naïve patients; *p* = 0.0013), and in those who underwent anatomic resection (versus wedge resection surgery; *p* = 0.012). Moreover, similar to the *WTS* cohort, the OS in the *TMA* cohort was also significantly longer in patients with low ASCL1 (*p* = 0.027; Figure 5A and supplementary material, Table S5) and high POU2F3 (*p* = 0.017; Figure 5C and supplementary material, Table S5)‐expressing tumors. Yet there was no statistically significant difference in OS with regard to NEUROD1 expression in the *TMA* cohort (*p* = 0.89; Figure 5B and supplementary material, Table S5). In the Cox multivariate model adjusted for clinicopathological variables in the *TMA* cohort (Figure 5), adjuvant CHT remained an independent prognostic factor for OS (*p* = 0.03), and moreover, low ASCL1 expression was associated with a tendency for better survival (hazard ratio [HR]: 0.67; *p* = 0.22).

### Proteomic profiling and cell viability assays of human SCLC cell lines reveal distinct vulnerability profiles defined by transcription regulators

**Figure 6:**
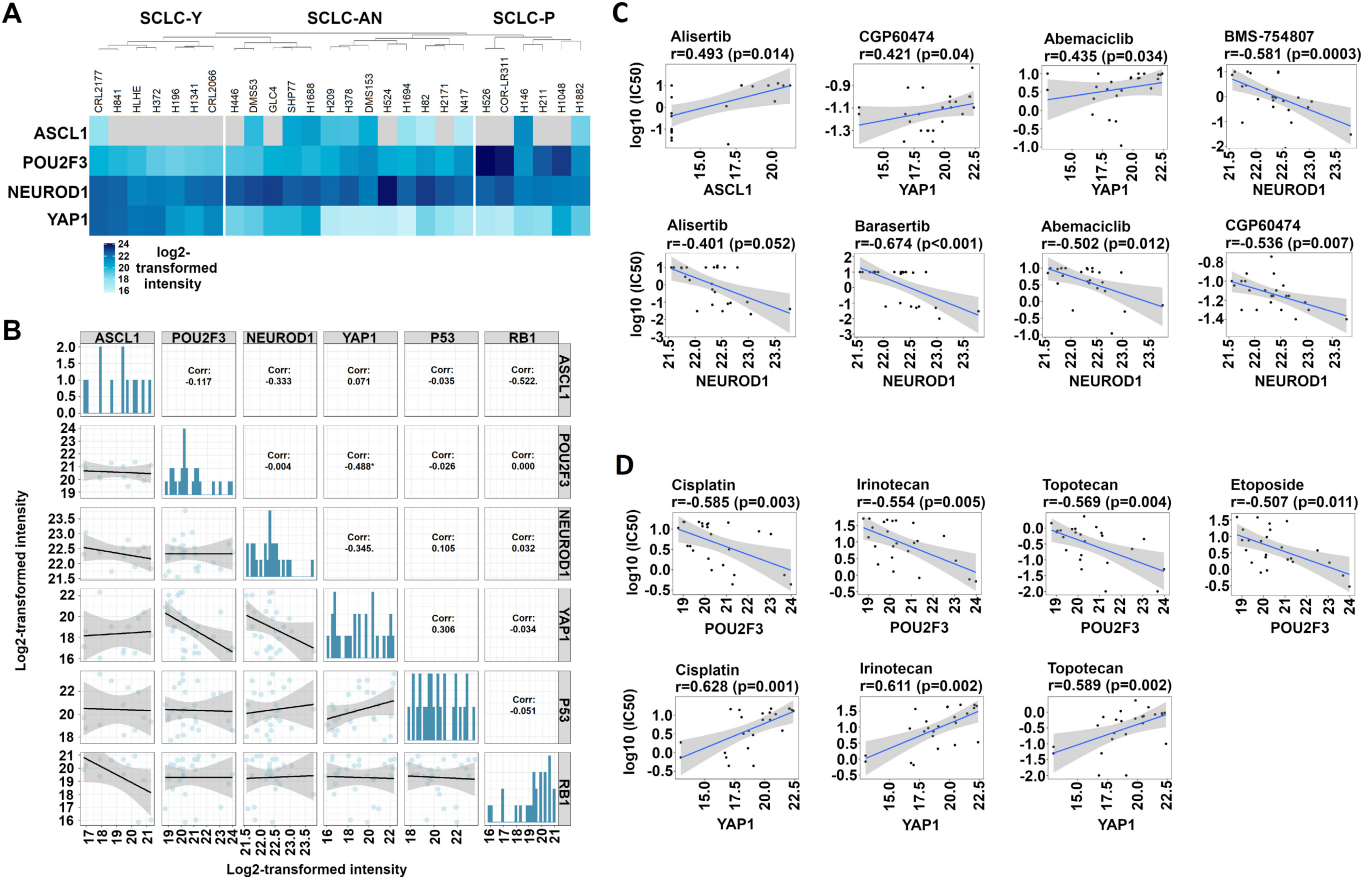
Proteomic profiling and *in vitro* efficacy of targeted and cytostatic drugs according to subtype‐specific proteins. (A) Unsupervised clustering of the investigated SCLC cell lines revealed a distinct YAP1‐driven, a mixed SCLC‐AN, and a heterogenous SCLC‐P cluster. The color bar represents the log_2_‐transformed protein intensity scores of ASCL1, NEUROD1, POU2F3, and YAP1. (B) Except for a statistically significant negative linear correlation between POU2F3 and YAP1 (*R* = −0.488), no significant correlation was identified between subtype‐specific and P53 and RB1 proteins. (C) Scatterplots demonstrating significant positive linear correlations between ASCL1 abundance and alisertib IC_50_ values (*r* = 0.493), and between YAP1 abundance and IC_50_ values of abemaciclib and CGP60474 (*r* = 0.435 and *r* = 0.421, respectively). Significant negative linear correlations between NEUROD1 proteomic abundance and IC_50_ values of alisertib (*r* = −0.401), barasertib (*r* = −0.674), abemaciclib (*r* = −0.502), CGP60474 (*r* = −0.536), and BMS‐754807 (*r* = −0.581) were also revealed. (D) Statistically significant negative linear correlations were found between POU2F3 abundance and IC_50_ values for cisplatin (*r* = −0.585), irinotecan (*r* = −0.554), topotecan (*r* = −0.569), and etoposide (*r* = −0.507). YAP1 abundance positively correlated with IC_50_ values for cisplatin (*r* = 0.628), irinotecan (*r* = 0.611), and topotecan (*r* = 0.589).

Our in‐depth proteomic analysis identified and quantitated more than 8,000 proteins in each of the 26 human SCLC cell lines. Interestingly, unsupervised clustering of samples based on protein abundance levels of ASCL1, NEUROD1, POU2F3, and YAP1 differentiated a distinct YAP1‐driven, a mixed SCLC‐AN, and a heterogenous SCLC‐P cluster (Figure 6A). As for the correlation pattern of subtype‐specific protein and RB1/P53 expressions (Figure 6B), we found a statistically significant negative linear correlation between expression of YAP1 and POU2F3 (*r* = −0.488), but no correlation with RB1 and P53. Next, in order to investigate the therapeutic relevance of subtype‐specific protein expressions, we correlated their proteomic abundance with the IC_50_ values of various targeted and chemotherapeutic agents [3, 4, 10] (supplementary material, Figure S6). As shown in Figure 6C, we found a statistically significant correlation between ASCL1 abundance and the IC_50_ values of the AURK‐inhibitor alisertib (*r* = 0.493) and, moreover, between YAP1 abundance and the sensitivities against the CDK‐inhibitors abemaciclib and CGP60474 (*r* = 0.435 and *r* = 0.421, respectively). Furthermore, we observed that high NEUROD1 proteomic abundance confers *in vitro* sensitivity to alisertib (*r* = −0.401), the AURK‐inhibitor barasertib (*r* = −0.674), abemaciclib (*r* = −0.502), CGP60474 (*r* = −0.536), and the IGF‐1R‐inhibitor BMS‐754807 (*r* = −0.581) (Figure 6C). As for standard‐of‐care chemotherapeutics (Figure 6D), we found a statistically significant negative linear correlation between POU2F3 abundance and IC_50_ values for cisplatin (*r* = −0.585), irinotecan (*r* = −0.554), topotecan (*r* = −0.569), and etoposide (*r* = −0.507). Furthermore, we found a statistically significant positive linear correlation between YAP1 abundance and IC_50_ values for cisplatin (*r* = 0.628), irinotecan (*r* = 0.611), and topotecan (*r* = 0.589).

## Discussion

Comprehensive genomic profiling in recent years has led to the refinement of SCLC classification by schemes defined by distinct gene expression profiles [4, 8]. The transcriptional landscape of a tumor, however, does not necessarily correspond with its protein‐level features. Although the emerging molecular classifications might serve as a framework for subtype‐specific therapy, many gaps in the assessment of SCLC remain and further investigations are required to define the expression patterns of the subtype‐defining markers at the protein level [4, 12, 18, 21, 22, 29, 30]. Additionally, due to the scarce availability of SCLC tissue specimens and the lack of appropriate clinical data, the extent to which different predominant subtypes influence the clinical outcomes in SCLC patients also warrants further clarification. Therefore, in this multicenter study we investigated the IHC expression and clinicopathological significance of key SCLC transcription regulators and RB1 and P53 proteins in a large cohort of surgically resected SCLC samples. Moreover, to identify potential subtype‐specific therapeutic vulnerabilities, we conducted an MS‐based proteomics study combined with *in vitro* cytotoxicity assays in a large panel of human SCLC cell lines.

Heterogeneity is prominent in SCLC tumors in terms of molecular diversity and NE features [6, 15]. Therefore, the dominant molecular subtype may be more evident in surgical samples than in small biopsies [18]. Additionally, in the case of small transbronchial or mediastinal biopsy specimens, crush artifacts may also be present [31]. In our study, performed on surgically resected SCLC specimens, the dominant molecular subtypes were SCLC‐A and SCLC‐QN in both investigated cohorts. ASCL1‐dominant SCLC‐A tumors (i.e. tumors with the ‘*classic*’ subtype [1, 32]) represent the vast majority of SCLCs and are generally associated with ‘*typical*’ SCLC morphology and high expression of NE markers [1]. However, we also found that a subset of SCLC‐A tumors coexpresses NEUROD1 and thus (in line with the findings of Baine *et al* [12]) that a combined SCLC‐AN subtype also exists. In support of this, recent genetically engineered mouse SCLC models suggest that temporal evolution from one molecular subtype to another (e.g. the transition from SCLC‐A to SCLC‐N) might be possible [10, 11, 16].

In our current study, we found no distinct SCLC‐Y subtype, which is consistent with the findings of Baine *et al* [12]. Instead, we identified a unique SCLC‐QN subtype characterized by the low expression of all four investigated transcription factors. Notably, SCLC‐QN is not defined by YAP1 expression, distinguishing our classification from the one proposed by Rudin *et al* [4]. Nevertheless, our results draw attention to the recently proposed SCLC‐I subtype, which is also defined by the low expression of all subtype‐specific markers [15]. SCLC‐I exhibits mesenchymal characteristics and an inflamed phenotype, thus capturing several features that are predictive of immune checkpoint blockade response in other tumors [15, 33, 34].

As for tumoral diversity, two forms of intratumoral heterogeneity were detected in our study. While some tumors had both subtype‐specific marker expressing and nonexpressing cells within the same areas, in other specimens, clusters of these cells were found in spatially distinct regions. This latter phenotype corresponds with the findings of Gay *et al* [15] and supports the idea that small biopsies might indeed not mirror the expression profile of the entire tumor. It is also important to mention here that given the high plasticity rate of SCLC [4], surgically resectable tumors might not completely mirror the clinicopathological features of the full SCLC disease spectrum. Therefore, our results concerning both the prognostic relevance and distribution pattern of molecular subtypes should be primarily considered in surgically treatable SCLC. Nevertheless, our findings might lay the framework for future validating studies in advanced‐stage patients as well.

With regard to the correlation pattern of subtype‐specific and P53 and RB1 proteins, we found a positive linear correlation between expression of YAP1 and RB1. This is in line with a recent preclinical study suggesting that YAP1‐expressing SCLC cell lines might be associated with intact *RB1* [14]. Nevertheless, given the low expression of both YAP1 and RB1, all results concerning these proteins should be interpreted with caution.

Due to the low number of surgical cases with appropriate clinical data and to the morphological heterogeneity (i.e. combined SCLC/NSCLC or SCLC/large cell neuroendocrine carcinoma) of tissue samples, the clinicopathological and prognostic relevance of subtype‐defining proteins remains incompletely elucidated. Our study is among the first to report the highly distinct prognostic relevance of molecular subtypes in surgically‐treated SCLC patients. In the current study, the highest OS rates were associated with non‐NE (SCLC‐P and SCLC‐QN), whereas the lowest with NE (SCLC‐A, SCLC‐N, SCLC‐AN) subtypes. In line with this, we found that in our univariate models the individual (i.e. subtype‐independent) expression of ASCL1 and POU2F3 expressions were associated with impaired and improved survival outcomes, respectively. Indeed, high‐grade NE features have been described as a sign of poor prognosis in lung cancer patients [35, 36]. Moreover, a recent IHC‐based analysis also suggests that patients with ASCL1‐positive tumors tend to have worse survival outcomes than those with ASCL1‐negative SCLCs [37]. Likewise, it has been reported that ASCL1 expression is a sign of poor prognosis in lung adenocarcinomas with NE differentiation [38]. Notably, in the current study high ASCL1 expression was also associated with late‐stage SCLC. Importantly, however, multivariate survival analysis revealed that ASCL1 expression influenced the survival outcomes independently from disease stage and other clinical factors.

A clear mechanistic link between NE features and survival, however, is yet to be elucidated in SCLC patients. Nevertheless, by using cases from the current *TMA* cohort, our group previously found that NE‐low SCLCs are associated with increased immune cell infiltration (i.e. CD45^+^, CD3^+^, and CD8^+^ cells) as compared to NE‐high tumors [39]. This is in line with the findings of Gazdar *et al* [40], who suggested that NE‐low tumors have increased immunogenicity and, therefore, ‘hot’ or ‘immune oasis’ phenotype as compared to NE‐high tumors with an ‘immune desert’ phenotype.

Of note, besides the bleak immunological landscape, another possible explanation for the poor survival outcomes in patients with NE‐high SCLC might be that these tumors are also associated with excessive hormone production, and thus with a higher rate of paraneoplastic syndromes [41, 42]. These paraneoplastic syndromes worsen the OS both in early‐ and late‐stage SCLC patients [43].

POU2F3‐driven SCLC tumors do not express classical NE lineage markers but express markers of the tuft cell lineage [13]. Therefore, reflecting on the lack of NE features, patients with SCLC‐P tumors supposedly have a better prognosis than those with NE SCLC. In support of this, POU2F3 immunostaining was associated with higher median OS in our univariate analysis. Our data concerning the prognostic relevance of POU2F3 are also partly in line with the findings of Huang *et al* [13]. In their study, those authors reassessed the RNA‐seq data from a previously published dataset [8] and found that patients with POU2F3‐high tumors exhibit nonsignificantly higher OS rates than those with POU2F3‐low tumors [13]. Notably, although we found no significant associations between disease stage and POU2F3 expression, patients in the POU2F3‐high subgroup tended to have early‐stage tumors. This might also contribute to the improved survival outcomes seen in these patients.

Despite the recent progress in our understanding of the molecular underpinnings of SCLC, the in‐depth proteomic characteristics of human SCLC still represents an area of active investigation [15, 44, 45, 46]. In contrast to our present IHC findings, unsupervised clustering of ASCL1, NEUROD1, POU2F3, and YAP1 proteins differentiated a distinct YAP1‐driven subtype in human SCLC cell lines. Of note, this discrepancy between SCLC cell lines and tissue samples regarding YAP1 expression has been described [12, 15] and warrants further investigation. Nevertheless, potential explanations that lie behind YAP1 loss might be related to tumor microenvironment, *RB1* mutation status, and sensitivity to standard‐of‐care CHT [14, 47]. Specifically, recent preclinical models suggest that YAP1 expression might be prominent in CHT‐refractory cases harboring wildtype *RB1* [14, 47].

Our results concerning the sensitivity of NEUROD1‐high‐expressing cells to AURK‐ and CDK‐inhibitors are in line with previous studies [3, 4, 10]. Notably, these MYC‐driven NEUROD1‐high cells have increased aurora kinase activity, thus predicting the efficacy of both AURKA‐ and AURKB‐inhibitors [4, 10]. Additionally, MYC‐amplified SCLCs are also expected to be susceptible to CDK‐inhibitors by inhibiting the synthetic lethal targets of MYC [10, 48].

To date, little is known about the therapeutic efficacy of chemotherapeutic agents in the context of molecular subtypes. In line with our results, Ito *et al* found that the loss of YAP1 might be a promising predictor of CHT responses in SCLC [47]. Given the strong correlation between YAP1‐abundance and CHT‐resistance, YAP1‐positive cell populations might indeed be more prominent in patients already treated with CHT, thus explaining the lack of the YAP1‐driven subtype in our surgically treated cohort. Finally, the increased sensitivity to cisplatin, irinotecan, topotecan, and etoposide of high POU2F3‐expressing cells might partly explain the improved survival outcomes seen in this molecular subtype. Of note, Gay *et al* also found a statistically nonsignificant tendency towards improved cisplatin response in SCLC‐P cell lines [15].

Our study has certain limitations due to its partly retrospective nature. First, patients in the *TMA* cohort were included over a long time‐period. Therefore, clinicopathological data were not available for some cases. Additionally, although most antigens in FFPE blocks are well preserved over time [49, 50], decreasing nuclear immunosignal intensity might occur in some older blocks. Of note, however, we obtained positive staining with all antibodies used for quality check (i.e. Bcl‐2 [23, 24], Ki‐67 [26, 27], SYP [28], and INI1 [25]) even in SCLC‐QN cases. Nevertheless, the weaker than expected staining rates with Ki‐67 suggest that, although our TMAs had proper quality, a reduction of immunosignal intensity might also occur in some cases. Even though the older TMA samples represent valuable assets to verify the findings of the *WTS* cohort concerning especially the prognostic relevance of subtype‐defining proteins and the presence of the SCLC‐QN subtype, they might not offer the same quality as the more recently prepared TMAs or the *WTS* specimens. Second, expression patterns on TMA samples might be also biased by intratumoral heterogeneity. However, to partly overcome this issue all of our TMAs contained two separate tissue cores from each patient. Notably, none of these limitations applied to the *WTS* cohort. Third, in our study SCLC‐QN was defined as a subset characterized by low expression of all four transcription regulators. However, in order to facilitate diagnosis, positive diagnostic markers are also needed for SCLC‐QN. In a recent study, Gay *et al* [15] refers to these ubiquitously negative tumors as SCLC‐I and suggests that mesenchymal factors may serve as positive, confirmatory markers to define this unique subset. Whether SCLC‐QN is identical with SCLC‐I and can be diagnosed by mesenchymal markers such as vimentin and AXL needs to be investigated in future studies. Finally, although the threshold values used for dichotomization were selected based on widely implemented diagnostic cutoffs, further studies are needed to confirm their accuracy in everyday practice.

By investigating the prognostic relevance and tissue distribution of subtype‐specific proteins in surgically treated SCLC patients, the current multicenter study attempts to fill a knowledge gap in our understanding of SCLC. We validated the new molecular subtype classification using a large cohort of surgical specimens and, moreover, found that differential expression of ASCL1, NEUROD1, and POU2F3 defines unique SCLC subtypes. However, our IHC analyses did not distinguish a specific YAP1‐driven subtype. Instead, we report an SCLC‐QN subtype accompanied by low expression of all four SCLC transcription regulators. Furthermore, we also showed that high POU2F3 expression is associated with improved median OS in a univariate analysis, whereas high ASCL1 expression is an independent negative prognosticator in surgically treated SCLC. Finally, our proteomic analyses of SCLC cell lines provided insight into specific correlation patterns between transcription regulators and the therapeutic efficacy of targeted and CHT agents. Altogether, our results might help in the development of subtype‐specific therapeutic approaches and follow‐up strategies in this devastating disease.

## Author contributions statement

ZM, NB, KS, VL and BD were responsible for conceptualization. ZM, OP, CL, BF, ZL, JM, MR, LB, JK, MK, JS, MM and GMV were responsible for data curation. ZM, AL and ZV were responsible for formal analysis. ZM, NB, AS, SP, KS, VL and BD were responsible for investigation. ZM, NB, KD, JF, BS, MR, HP, GMV, VL and BD were responsible for methodology. ZM, KD, JF, GG, BS, CR, FRH, HP, KB, FRV, MAH, TK, KH and BD were responsible for project administration. ZM and OP were responsible for software. ZM and OP were responsible for visualization. OP was responsible for validation. FO, GG, LB, HP, IK, MK, JS, MM, GMV, KB, FRV, MAH, TK and KH were responsible for resources. KS, VL and BD were responsible for supervision. ZM and BD were responsible for writing ‐ original draft. All authors were responsible for writing ‐ review and editing.

## Supporting information


Supplementary materials and methods

Supplementary figure legends
**Figure S1.** Representative IHC images of specimens from the TMA cohort
**Figure S2.** Expression of subtype‐specific markers and P53 and RB1 in the WTS cohort
**Figure S3.** Kaplan–Meier estimates for OS in surgically resected SCLC patients according to basic clinicopathological characteristics in the WTS cohort
**Figure S4.** Kaplan–Meier estimates for OS in the WTS cohort according to NE subtypes
**Figure S5.** Kaplan–Meier curves for OS in surgically resected SCLC patients according to basic clinicopathological characteristics in the TMA cohort
**Figure S6.** Correlation between the proteomic abundances of subtype‐specific transcription factors and the *in vitro* efficacy of targeted and chemotherapeutic agents

**Table S1.** Antibodies used for immunohistochemistry

**Table S2.** Clinicopathological characteristics of the WTS cohort

**Table S3.** Clinicopathological characteristics of the TMA cohort

**Table S4.** Prognostic impact of subtype‐specific markers and other relevant proteins in the WTS cohort

**Table S5.** Prognostic impact of subtype‐specific markers and other relevant proteins in the TMA cohort

## Data Availability

The data that support the findings of this study are available from the corresponding author upon reasonable request.
