# Supplementary material for: Expression patterns and prognostic relevance of subtype‐specific transcription factors in surgically resected small‐cell lung cancer: an international multicenter study
Source: J Pathol. 2022 May 25;257(5):674–86. doi: 10.1002/path.5922 (PMC9541929; doi:10.1002/path.5922)
Supplement: Supplementary file 1 — Supplementary materials and methods [file PATH-257-674-s007.docx]

**Expression patterns and prognostic relevance of subtype-specific transcription factors in surgically resected small cell lung cancer: an international multicenter study**

Z Megyesfalvi, N Barany *et al. J Pathol* DOI: 10.1002/path.5922

**Supplementary materials and methods**

Reference numbers refer to the main text list

*Study population*

In this multicenter study, we included 386 patients with histologically confirmed SCLC who underwent surgical resection in the following five Central European medical centers: National Koranyi Institute of Pulmonology (Budapest, Hungary), National Institute of Oncology (Budapest, Hungary), Medical University of Vienna (Vienna, Austria), Medical University of Graz (Graz, Austria) and University Clinic for Respiratory and Allergic Diseases Golnik (Golnik, Slovenia). Both elective cases (that were known to be SCLC before the surgical resection) and incidental cases (diagnosed after surgery based on the resection specimen) were included. Because previous studies have uncovered substantial intratumoral heterogeneity in the expression pattern of subtype-specific SCLC markers [15,18], we grouped our patients either into a *whole tissue section (WTS) cohort* where complete surgical formalin-fixed paraffin-embedded (FFPE) blocks were available (n=141) or a *TMA cohort* (tissue microarray cohort, n=247) and analyzed these two cohorts separately. Clinical data regarding age, gender, smoking status, pathological stage, and survival were retrospectively collected from medical records and/or records from the National Health Insurance Offices or Central Statistical Offices of each participating country. The study was conducted in accordance with the guidelines of the Helsinki Declaration of the World Medical Association and with the approval of the national level ethics committee of each participating country. Due to the retrospective nature of the study, the requirement for written informed consent was waived. After clinical information was collected, patient identifiers were removed, and subsequently, patients could not be identified either directly or indirectly.

*Treatment*

Diagnostic and therapeutic approaches were conducted in accordance with the contemporary National Comprehensive Cancer Network (NCCN) guidelines presenting no differences across the host institutes[51]. All patients underwent lung resection surgery by either anatomic or wedge resection between 2000 and 2020 (*WTS cohort*) or 1978 and 2016 (*TMA cohort*). When recommended, adjuvant chemotherapy (CHT) consisted of a platinum-etoposide doublet regimen or a combination of cyclophosphamide, epirubicin, and vincristine (CEV). None of the included patients received immunotherapy.

*Whole tissue sections and TMA specimens of surgically resected SCLC tumors*

All SCLC tumor tissue specimens were obtained by surgical resection. The FFPE tissue samples were processed and examined for routine diagnostic workup. This was performed by board-certified pathologists of the host institute according to the contemporary diagnostic guidelines and by using specific IHC stains [28]. In addition, prior to enrolment, all slides were re-evaluated by a pathologist to confirm the diagnosis of SCLC. Of note, only pure SCLC (i.e. no combined SCLC/NSCLC or SCLC/large cell neuroendocrine carcinoma) cases were included. With regards to the TMA cohort, TMA construction was performed at the University of Colorado Denver (Aurora CO, USA), as previously described [39,52]. In brief, the H&E slides were reviewed by a pathologist to select the most viable tumor areas. Marked-stained sections were used to guide the technician as to the location for punch tissue removal. Two 1.0 mm cores of tissue were taken from each donor tissue.

*Immunohistochemistry*

All tissue samples were analyzed for expression of the four markers of SCLC subtypes (ASCL1, NEUROD1, POU2F3 and YAP1) as well as for P53 and RB1 [7]. The antibodies and the IHC protocols are summarized in supplementary material, Table S1. In brief, after deparaffinization and rehydration of the 4 µm-thick sections, the slides were heated for 20 min in 10 mM citrate buffer (pH 6.0) in a pressure cooker. NEUROD1, P53 and RB antibody labeling represented an exception where 10 mM Tris-EDTA (pH 9.0) was used in this step. The slides were incubated in a 0.3% H_2_O_2_ solution for 10 min in order to reduce the nonspecific background staining. Signal amplification was then performed according to the manufacturer’s recommendations of Novolink^TM^ Polymer Detection System kit from Leica Biosystems (RE7150-K, Wetzlar, Germany), followed by antibody incubation for 1 h at room temperature (except in the case of POU2F3 antibody which was incubated overnight at 4 °C). Antibody binding was detected by using ImmPACT DAB Substrate Kit from Vector Laboratories. Nuclei were counterstained using hematoxylin. All antibodies were validated by using appropriate tissue controls. Expression of the given marker was examined blinded to clinical data by two experienced independent pathologists. Slides were examined with 20× and 40× objective lenses, and the staining index (percentage of all tumor cells showing positive staining) was determined. For certain analyses, tumor samples were dichotomized into low versus high expressing subgroups according to the IHC expression levels. To bring the obtained results closer to everyday practice, the cut-off values were defined based on the median protein expression and on the generally used diagnostic thresholds. Accordingly, after defining the median expression level of each protein (supplementary material, Figure S2), the nearest commonly used pathological threshold (e.g. 1%, 5%, 10%, or 50%) was selected as cut-off. Accordingly, the following threshold values were used for dichotomized expression levels: 50% and 5% for ASCL1, 5% for NEUROD1, 1% for POU2F3, positivity (>0%) for YAP1, 50% for P53 and positivity (>0%) for RB1. Of note, for ASCL1, separate cut-off values were used in the *WTS-* and *TMA cohorts* due to weaker staining of these olderTMA samples. To assess the quality and reliability of the older FFPE blocks in the *TMA cohort*, all included TMAs were also stained with antibodies against Bcl-2 [23,24], Ki-67 [26,27], SYP [28] and INI1 [25].

*Mass spectrometry-based proteomic analyses*

In-depth proteomic analysis was conducted as described previously [53]. In brief, after digestion in S-Trap™ 96-well Plate format (ProtiFi, Farmingdale, NY, USA), peptides were analyzed by nanoscale liquid chromatography separation combined with tandem mass spectrometry (nLC-MS/MS) using label-free quantification. The nLC-MS/MS analysis was performed on an Ultimate 3000 RSLC nano pump (Thermo Fisher Scientific, Waltham, MA, USA) coupled to a Q-Exactive HF-X (Thermo Fisher Scientific) mass spectrometer equipped with an EASY-Spray ion source. Raw files were searched in Proteome Discoverer 2.4 (Thermo Fisher Scientific) using Spectral Library (Proteome tools human spectrum library v.12/11/2019; URL: http://www.proteometools.org/index.php?id=home) and SEQUEST HT searches against UniProtKB human database (URL: https://www.uniprot.org/proteomes/UP000005640; downloaded: 15/01/2019). The raw protein intensities were then log_2_-transformed and the samples were median-normalized by centering all the samples to the global median. Triplicate measurements were averaged, followed by a filter for minimum 80% valid values and left-censored missing data imputation.

*Cell viability assay*

Cells were seeded in 100 µl growth medium into 96-well plates and on the next day treated with 100 µl medium containing drugs (MedChemExpress, Monmouth Junction, NJ, USA) as indicated. After 72h, cell viability was measured using the EZ4U MTT assay (Biomedica, Vienna, Austria) according to the manufacturer’s protocol. All experiments were performed at least 3 times in triplicate. IC_50_ values were calculated from dose response curves ranging from 0–100 µM normalized to the vehicle-treated control. The following substances were used: alisertib and barasertib (AURK-inhibitors), BMS-754807 and picropodophyllin (PPP) [IGF-1R-inhibitors], abemaciclib and CGP60474 (CDK-inhibitors), cisplatin, etoposide, topotecan, epirubicin and irinotecan (chemotherapeutics).

*Statistical analyses*

All statistical analyses were performed using R version 3.6.3 (R Foundation for Statistical Computing, Vienna, Austria). Categorical and ordinal parameters including the dichotomized IHC expression levels and clinicopathological characteristics were statistically analyzed by χ^2^ test or Fisher's exact test. Hierarchical clustering of samples based on the measured expression levels was performed with the ComplexHeatmap R package (v.2.10.0; URL: https://jokergoo.github.io/ComplexHeatmap-reference/book/; date last accessed: 5/10/2021). The distance matrix was calculated using Euclidean distance measure and the dendrograms were created using the complete-linkage clustering method. Correlations of expression levels were calculated in a pairwise manner using Pearson correlation. The value of linear correlation coefficient (r) varies from −1 to 1 both values inclusive. Of note, in case of proteomic data, the Benjamini–Hochberg correction was used to adjust for multiple testing. Survival curves were estimated by Kaplan–Meier plots and the differences between the groups were compared using the log‐rank test. Median follow‐up time was estimated using the reverse‐censored Kaplan–Meier method. Variables where the log-rank p-values were equal to or below 0.25 were selected for multivariate analysis. Multivariate analysis was performed using a Cox regression model. The proportional hazards assumption was verified by calculating the correlation coefficient between transformed survival time and the scaled Schoenfeld residuals using cox.zph() function of the survival R package, and checking if any of the correlations are significant (***, p<0.001; **, p<0.01; *, p<0.05; †, p<0.10).
