# Supplementary material for: Expression patterns and prognostic relevance of subtype‐specific transcription factors in surgically resected small‐cell lung cancer: an international multicenter study
Source: J Pathol. 2022 May 25;257(5):674–86. doi: 10.1002/path.5922 (PMC9541929; doi:10.1002/path.5922)
Supplement: Supplementary file 2 — Supplementary figure legends Figure S1. Representative IHC images of specimens from the TMA cohort Figure S2. Expression of subtype‐specific markers and P53 and RB1 in the WTS cohort Figure S3. Kaplan–Meier estimates for OS in surgically resected SCLC patients according to basic clinicopathological characteristics in the WTS cohort Figure S4. Kaplan–Meier estimates for OS in the WTS cohort according to NE subtypes Figure S5. Kaplan–Meier curves for OS in surgically resected SCLC patients according to basic clinicopathological characteristics in the TMA cohort Figure S6. Correlation between the proteomic abundances of subtype‐specific transcription factors and the in vitro efficacy of targeted and chemotherapeutic agents [file PATH-257-674-s001.zip › path5922-sup-SuppFigLegs.docx]

**Expression patterns and prognostic relevance of subtype-specific transcription factors in surgically resected small cell lung cancer: an international multicenter study**

Z Megyesfalvi, N Barany *et al. J Pathol* DOI: 10.1002/path.5922

**Supplementary figure legends S1–S6**

**Figure S1. Representative IHC images of specimens from the TMA cohort.** IHC for (A) Bcl-2, (B) SYP, (C) Ki-67, and (D) INI1.

**Figure S2. Expression of subtype-specific markers and P53 and RB1 in the WTS cohort.** (A) ASCL1, (B) NEUROD1, (C) POU2F3, (D) YAP1, (E) P53, (F) RB1.

**Figure S3. Kaplan–Meier estimates for OS in surgically resected SCLC patients according to basic clinicopathological characteristics in the *WTS cohort*.** Kaplan–Meier curves comparing OS of SCLC patients according to (A) gender, (B) smoking history, (C) COPD, as comorbidity, (D) diabetes, as comorbidity, (E) tumor localization*, (F) disease stage^#^, (G) surgery type, (H) intratumoral necrosis, (I) vascular invasion, (J) adjuvant CHT and (K) adjuvant radiotherapy. *Endoscopically visible primary SCLCs were defined as central, otherwise as peripheral tumors. ^#^Early-stage refers to stage I and II, whereas late-stage to stage III and IV SCLC. Abbreviations: OS = overall survival; CHT = chemotherapy.

**Figure S4. Kaplan–Meier estimates for OS in the *WTS cohort* according to NE subtypes.** SCLC tumors with non-NE subtype were significantly associated with improved OS (versus NE SCLCs; median OSs were 46 versus 28.8 months, respectively; P=0.003). Abbreviations: OS = overall survival; NE = neuroendocrine.

**Figure S5. Kaplan–Meier curves for OS in surgically resected SCLC patients according to basic clinicopathological characteristics in the *TMA cohort*.** Plots show the associations between OS and (A) gender, (B) smoking history, (C) COPD, as comorbidity, (D) diabetes, as comorbidity, (E) disease stage*, (F) surgery type, (G) intratumoral necrosis, (H) vascular invasion and (I) adjuvant CHT. *Early-stage was defined as stage I or II, whereas late-stage as stage III or IV SCLC. Abbreviations: OS = overall survival; CHT = chemotherapy.

**Figure S6. Correlation between the proteomic abundances of subtype-specific transcription factors and the *in vitro* efficacy of targeted and chemotherapeutic agents.** Plots showing correlation analyses between subtype-specific proteins (ASCL1, NEUROD1, POU2F3 and YAP1) and the IC_50_ values of abemaciclib, alisertib, barasertib, BMS-754807, picropodophyllin (PPP), CGP60474, cisplatin, epirubicin, etoposide, irinotecan and topotecan.
