# Supplementary figures and images for: Expression patterns and prognostic relevance of subtype‐specific transcription factors in surgically resected small‐cell lung cancer: an international multicenter study
Source: J Pathol. 2022 May 25;257(5):674–86. doi: 10.1002/path.5922 (PMC9541929; doi:10.1002/path.5922)

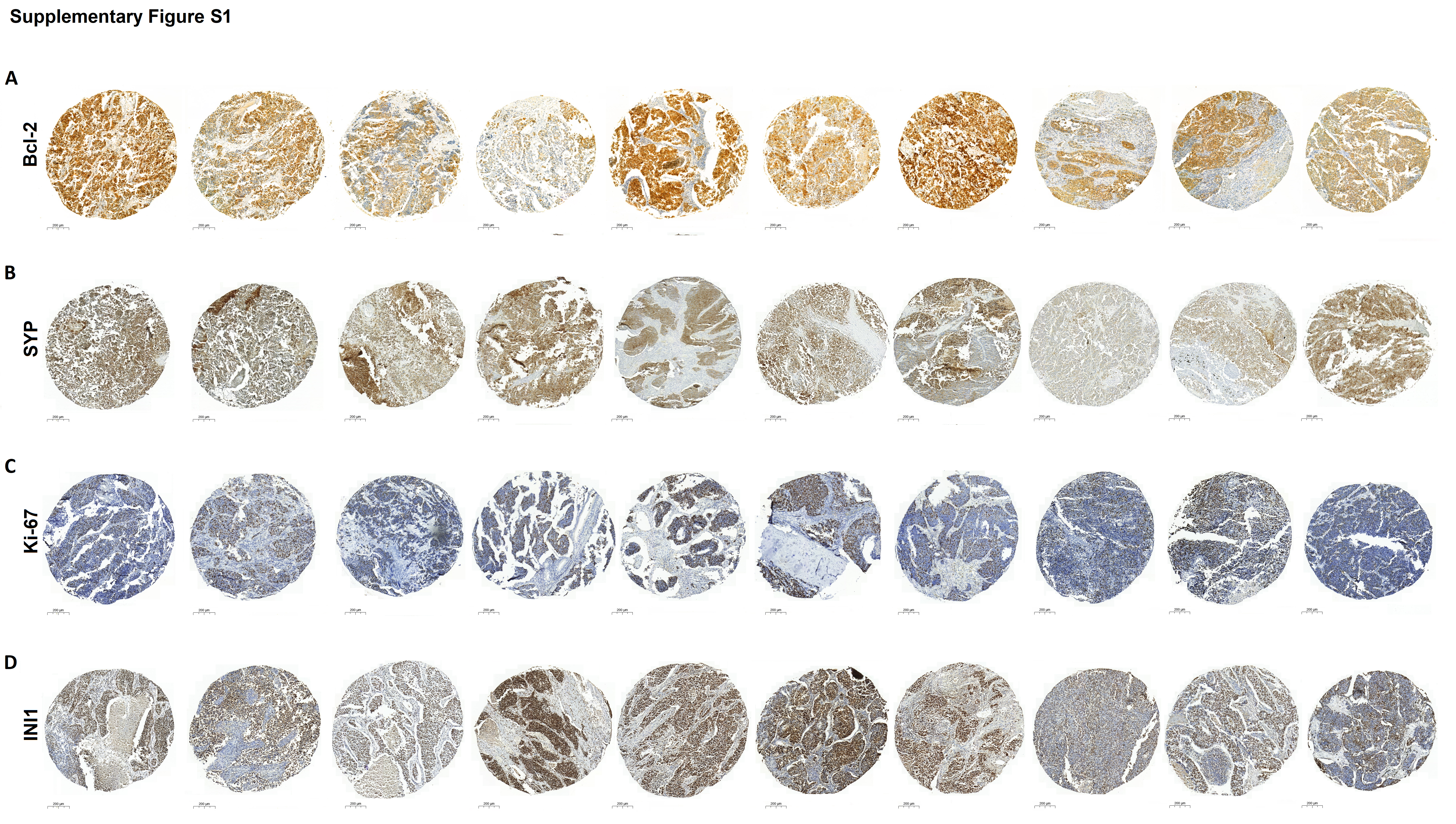

Supplement: Supplementary file 2 — Supplementary figure legends Figure S1. Representative IHC images of specimens from the TMA cohort Figure S2. Expression of subtype‐specific markers and P53 and RB1 in the WTS cohort Figure S3. Kaplan–Meier estimates for OS in surgically resected SCLC patients according to basic clinicopathological characteristics in the WTS cohort Figure S4. Kaplan–Meier estimates for OS in the WTS cohort according to NE subtypes Figure S5. Kaplan–Meier curves for OS in surgically resected SCLC patients according to basic clinicopathological characteristics in the TMA cohort Figure S6. Correlation between the proteomic abundances of subtype‐specific transcription factors and the in vitro efficacy of targeted and chemotherapeutic agents [file PATH-257-674-s001.zip › path5922-sup-FigureS1.tif]

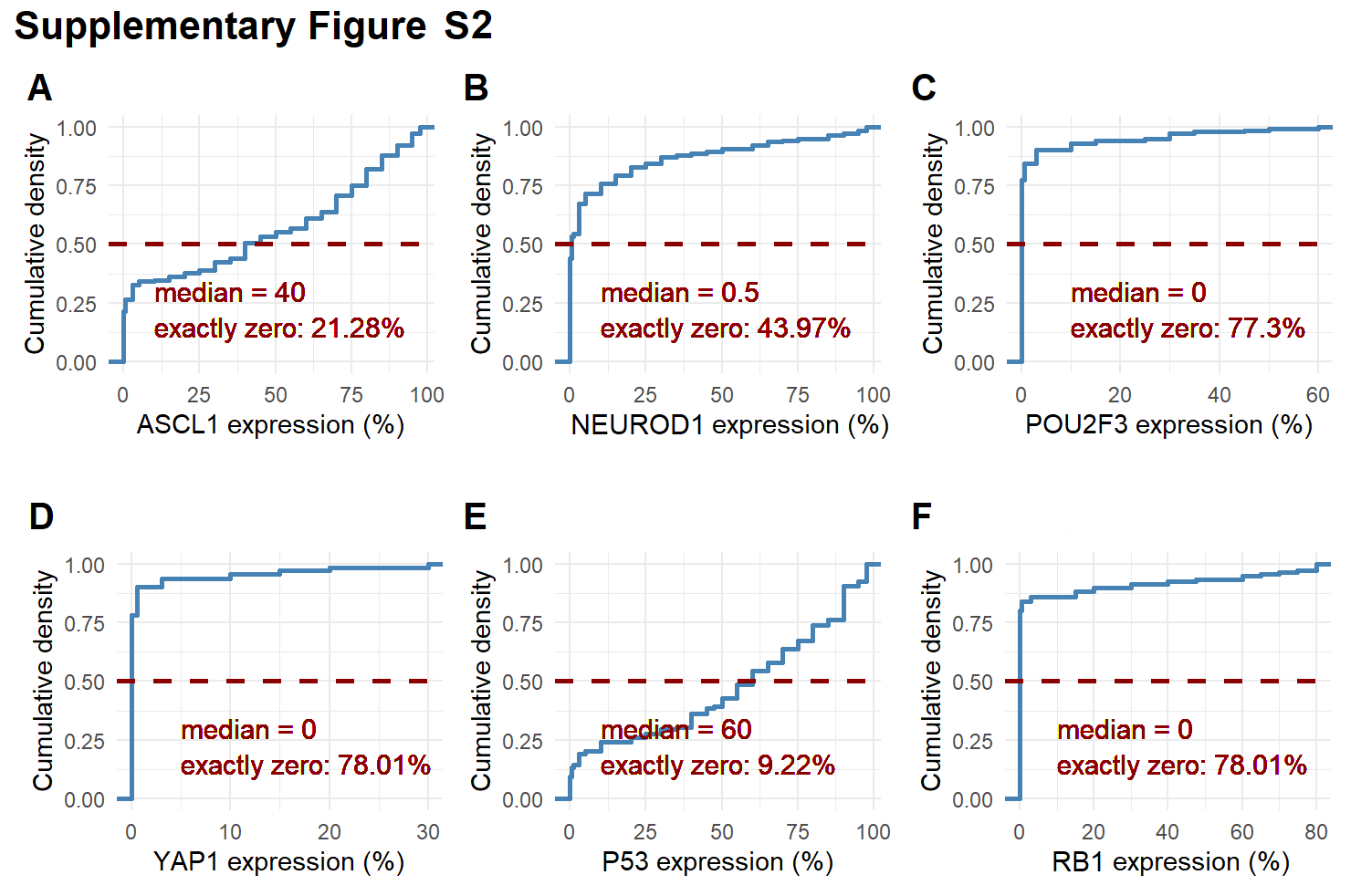

Supplement: Supplementary file 2 — Supplementary figure legends Figure S1. Representative IHC images of specimens from the TMA cohort Figure S2. Expression of subtype‐specific markers and P53 and RB1 in the WTS cohort Figure S3. Kaplan–Meier estimates for OS in surgically resected SCLC patients according to basic clinicopathological characteristics in the WTS cohort Figure S4. Kaplan–Meier estimates for OS in the WTS cohort according to NE subtypes Figure S5. Kaplan–Meier curves for OS in surgically resected SCLC patients according to basic clinicopathological characteristics in the TMA cohort Figure S6. Correlation between the proteomic abundances of subtype‐specific transcription factors and the in vitro efficacy of targeted and chemotherapeutic agents [file PATH-257-674-s001.zip › path5922-sup-FigureS2.tif]

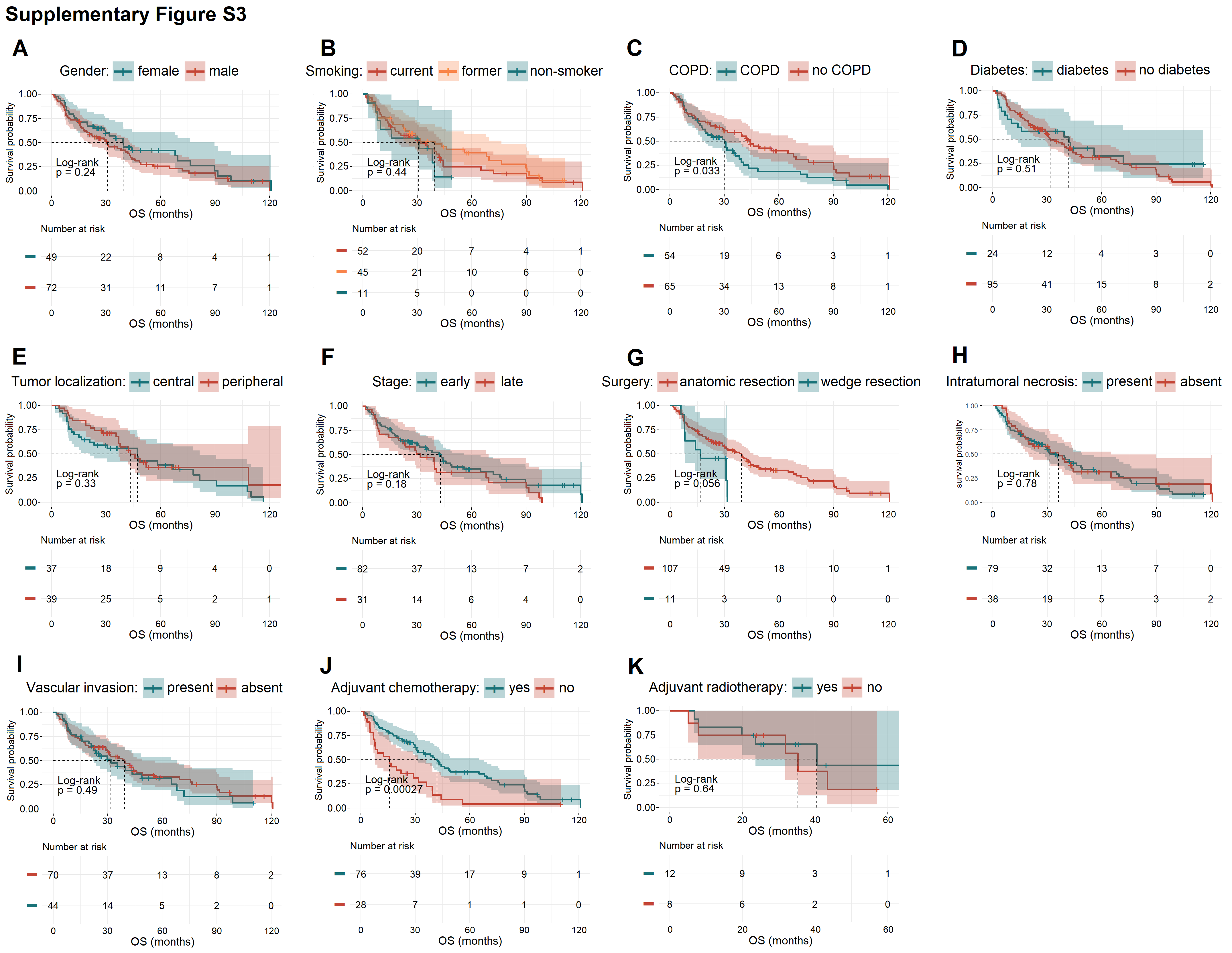

Supplement: Supplementary file 2 — Supplementary figure legends Figure S1. Representative IHC images of specimens from the TMA cohort Figure S2. Expression of subtype‐specific markers and P53 and RB1 in the WTS cohort Figure S3. Kaplan–Meier estimates for OS in surgically resected SCLC patients according to basic clinicopathological characteristics in the WTS cohort Figure S4. Kaplan–Meier estimates for OS in the WTS cohort according to NE subtypes Figure S5. Kaplan–Meier curves for OS in surgically resected SCLC patients according to basic clinicopathological characteristics in the TMA cohort Figure S6. Correlation between the proteomic abundances of subtype‐specific transcription factors and the in vitro efficacy of targeted and chemotherapeutic agents [file PATH-257-674-s001.zip › path5922-sup-FigureS3.tif]

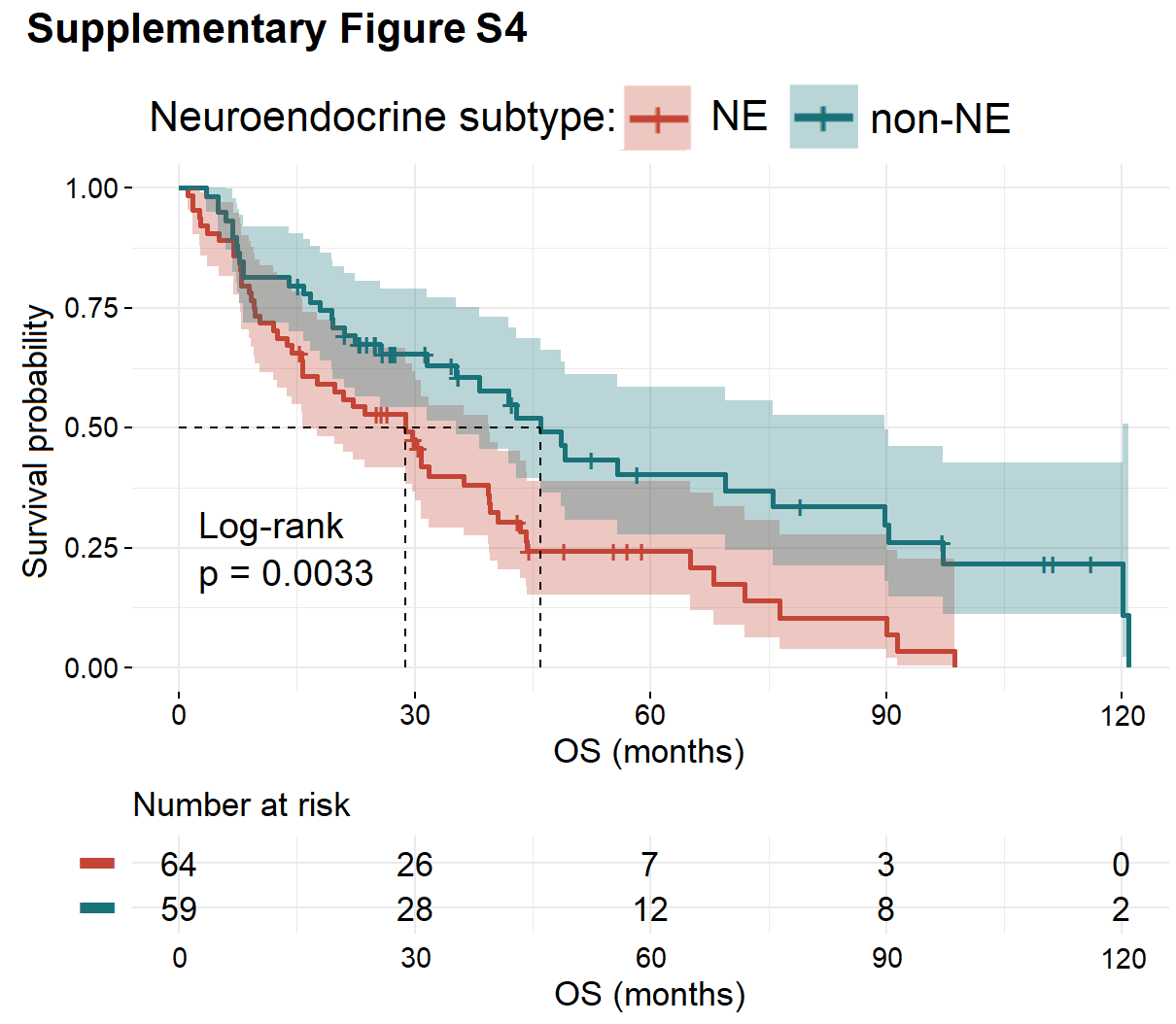

Supplement: Supplementary file 2 — Supplementary figure legends Figure S1. Representative IHC images of specimens from the TMA cohort Figure S2. Expression of subtype‐specific markers and P53 and RB1 in the WTS cohort Figure S3. Kaplan–Meier estimates for OS in surgically resected SCLC patients according to basic clinicopathological characteristics in the WTS cohort Figure S4. Kaplan–Meier estimates for OS in the WTS cohort according to NE subtypes Figure S5. Kaplan–Meier curves for OS in surgically resected SCLC patients according to basic clinicopathological characteristics in the TMA cohort Figure S6. Correlation between the proteomic abundances of subtype‐specific transcription factors and the in vitro efficacy of targeted and chemotherapeutic agents [file PATH-257-674-s001.zip › path5922-sup-FigureS4.tif]

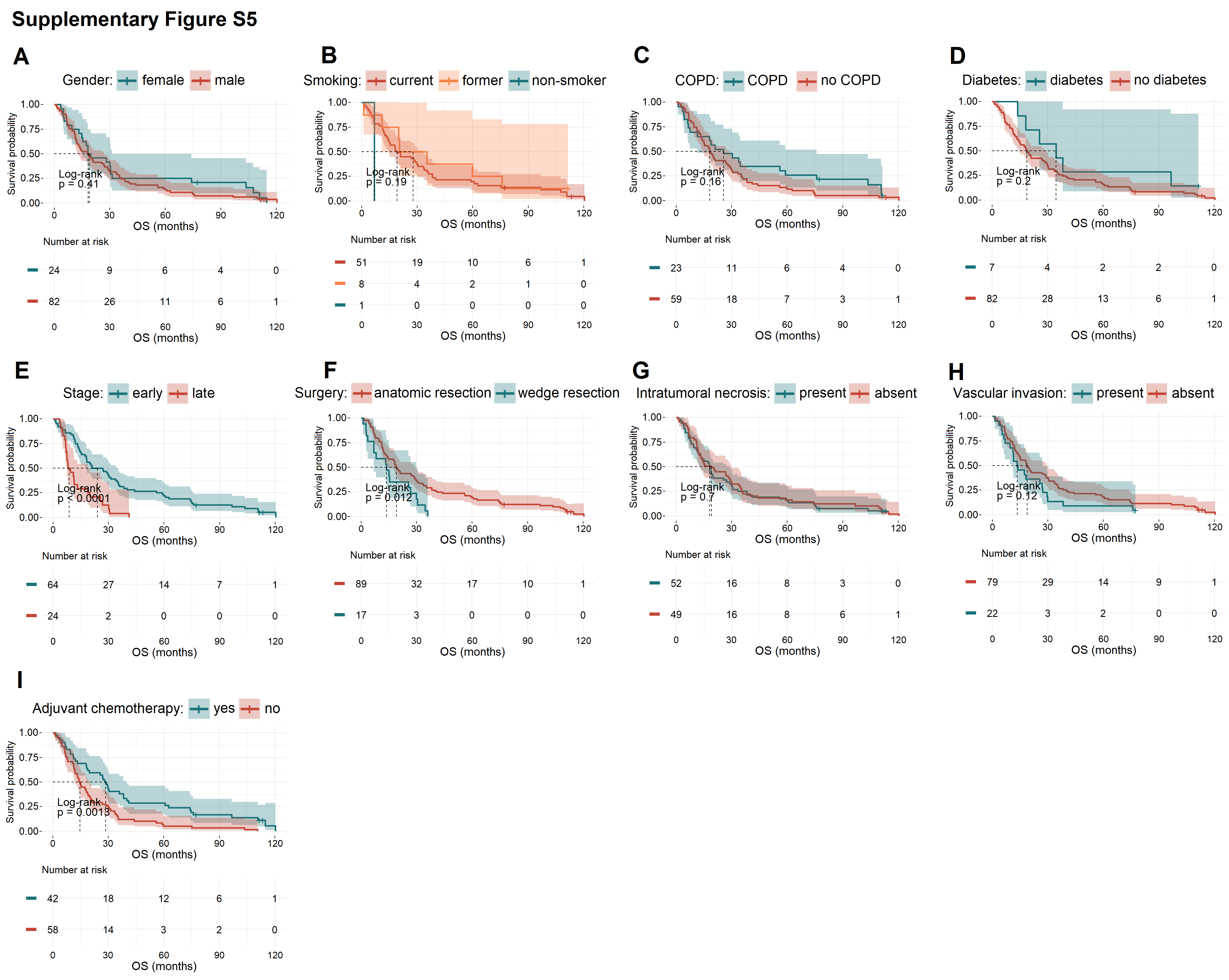

Supplement: Supplementary file 2 — Supplementary figure legends Figure S1. Representative IHC images of specimens from the TMA cohort Figure S2. Expression of subtype‐specific markers and P53 and RB1 in the WTS cohort Figure S3. Kaplan–Meier estimates for OS in surgically resected SCLC patients according to basic clinicopathological characteristics in the WTS cohort Figure S4. Kaplan–Meier estimates for OS in the WTS cohort according to NE subtypes Figure S5. Kaplan–Meier curves for OS in surgically resected SCLC patients according to basic clinicopathological characteristics in the TMA cohort Figure S6. Correlation between the proteomic abundances of subtype‐specific transcription factors and the in vitro efficacy of targeted and chemotherapeutic agents [file PATH-257-674-s001.zip › path5922-sup-FigureS5.tif]

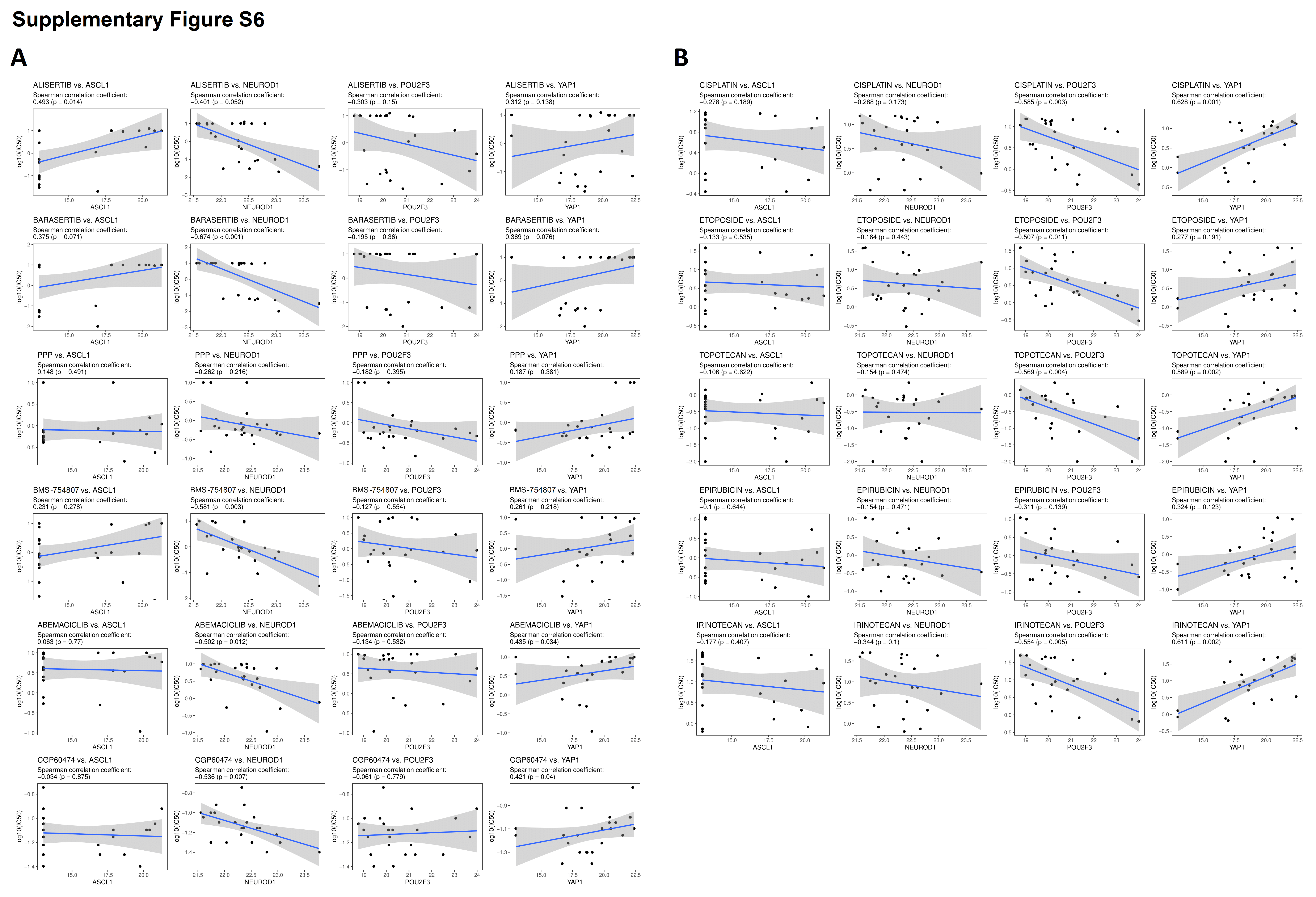

Supplement: Supplementary file 2 — Supplementary figure legends Figure S1. Representative IHC images of specimens from the TMA cohort Figure S2. Expression of subtype‐specific markers and P53 and RB1 in the WTS cohort Figure S3. Kaplan–Meier estimates for OS in surgically resected SCLC patients according to basic clinicopathological characteristics in the WTS cohort Figure S4. Kaplan–Meier estimates for OS in the WTS cohort according to NE subtypes Figure S5. Kaplan–Meier curves for OS in surgically resected SCLC patients according to basic clinicopathological characteristics in the TMA cohort Figure S6. Correlation between the proteomic abundances of subtype‐specific transcription factors and the in vitro efficacy of targeted and chemotherapeutic agents [file PATH-257-674-s001.zip › path5922-sup-FigureS6.tif]
